# When Tapering Is Not an Option: Transitioning From High-Dose Methadone to Buprenorphine

**DOI:** 10.7759/cureus.114081

**Published:** 2026-08-06

**Authors:** Sarah Petelinsek, Jarom Morris, Ethan Zaugg, Michael Coudreaut

**Affiliations:** 1 School of Medicine, University of Utah, Salt Lake City, USA; 2 School of Medicine, Noorda College of Osteopathic Medicine, Salt Lake City, USA; 3 Department of Psychiatry, Intermountain Healthcare, Salt Lake City, USA

**Keywords:** buprenorphine, case report, methadone, opioid use disorder, opioid withdrawal, qtc prolongation, ventricular arrhythmia

## Abstract

Methadone is a commonly used medication for opioid use disorder (OUD) but carries known risks, including corrected QT interval (QTc) prolongation and ventricular arrhythmias. Current guidelines recommend gradual methadone tapering or buprenorphine microdosing when transitioning to buprenorphine to minimize precipitated withdrawal. However, evidence guiding management when methadone must be urgently discontinued because of medical contraindications is limited.

We describe a 41-year-old woman with OUD receiving high-dose methadone who presented after cardiac arrest with marked QTc prolongation, hypokalemia, and suspected methadone-associated cardiotoxicity. Given the life-threatening risk of ventricular arrhythmias, methadone was discontinued without tapering. The patient was managed symptomatically with short-acting opioids for withdrawal and was subsequently initiated on buprenorphine approximately 60 hours after her last methadone dose, once moderate withdrawal had developed. Although she experienced transient worsening of withdrawal symptoms after induction, she stabilized within 48 hours and was discharged on maintenance buprenorphine with outpatient follow-up.

While gradual tapering remains standard practice, this case demonstrates that complete methadone cessation without tapering, followed by appropriately timed buprenorphine initiation, may be feasible when rapid discontinuation is medically necessary. Careful monitoring, shared decision-making, and symptomatic support remain essential.

## Introduction

Methadone, a synthetic full mu-opioid receptor agonist, is commonly used for maintenance treatment of opioid use disorder (OUD) [1,2]. While highly effective, methadone has a long half-life and is associated with dose-dependent corrected QT interval (QTc) prolongation, an electrocardiographic finding that reflects delayed ventricular repolarization and may increase the risk of life-threatening ventricular arrhythmias such as torsades de pointes [3,4]. Buprenorphine, a partial mu-opioid receptor agonist with high receptor affinity, carries a substantially lower risk of QTc prolongation but may precipitate opioid withdrawal if initiated before sufficient methadone clearance has occurred.

Current literature recommends that high-dose methadone be discontinued gradually using a tapering approach. The recommended taper rate is less than 10% of the established maintenance dose at intervals of 10-14 days, with some studies recommending tapers as slow as 3% of the dose per week [1,2,5]. Longer tapers have been associated with better patient outcomes, with a stepped, gradual taper involving dose reductions during only 25%-50% of weeks demonstrating the highest rates of success [6]. The greatest risks associated with abrupt methadone discontinuation are relapse and fatal overdose [1,2,5]. However, these risks remain present, although generally to a lesser extent, with gradual tapering as well [7].

Another significant concern when discontinuing methadone, particularly during transition to buprenorphine, is precipitated withdrawal [8]. Current practice guidelines and the literature recommend two major approaches to minimize this risk: a slow methadone taper with initiation of buprenorphine after the patient has reduced to less than 30 mg of methadone daily, or buprenorphine microdosing [9-13]. However, in situations in which methadone is contraindicated, such as QTc prolongation [9], there is a paucity of evidence to guide optimal management.

To our knowledge, this is the first reported case in which complete methadone cessation without tapering was successfully used to transition a patient to buprenorphine in the setting of life-threatening QTc prolongation.

## Case presentation

Patient information

This is a 41-year-old woman with a past medical history significant for alcohol use disorder and OUD who had been receiving high-dose methadone maintenance therapy for approximately four years. At the time of presentation, her maintenance dose was 90 mg twice daily. She initially presented to the emergency department via emergency medical services (EMS) after a seizure-like episode. EMS found her to be pulseless and initiated cardiopulmonary resuscitation (CPR). She was found to have a prolonged QTc interval of 652 ms without signs of ischemic changes. Her laboratory results were also significant for a potassium level of 2.9 mmol/L. The patient was also found to have high-anion-gap metabolic acidosis and pneumonia, most likely secondary to aspiration. She was admitted to the cardiac intensive care unit, where a psychiatry consultation was requested. She had a negative review of systems and denied any pertinent medical, surgical, or family history. She developed clinically significant opioid withdrawal symptoms during hospitalization that were managed symptomatically while awaiting buprenorphine induction. Because this report was prepared retrospectively and the complete inpatient medical record was no longer available, standardized withdrawal assessments (e.g., Clinical Opioid Withdrawal Scale (COWS) scores) and detailed symptom inventories could not be reported.

Clinical finding

The patient had been receiving methadone maintenance therapy for four years before admission and had been titrated to a maintenance dose of 90 mg twice daily. She also reported a history of alcohol use disorder but had remained abstinent from alcohol for several years. She reported using only oral opioids and had been in recovery for several years before this admission. She endorsed one recent relapse. She took her last dose of methadone approximately 12 hours before admission. For a full timeline of clinical events, see Table 1.

**Table 1:** Patient clinical timeline CPR, cardiopulmonary resuscitation; ROSC, return of spontaneous circulation; QTc, corrected QT interval; ICU, intensive care unit; ECG, electrocardiography

Timeline	Clinical events
Day 0 (pre-admission)	Patient receiving high-dose methadone (90 mg twice daily); last dose approximately 12 hours before presentation.
Day 0 (presentation)	Presented with seizure-like activity and was found pulseless. CPR initiated with return of spontaneous circulation (ROSC). ECG demonstrated QTc 652 ms; laboratory studies revealed hypokalemia (K 2.9 mmol/L). Admitted to the ICU. Methadone was discontinued due to concern for cardiotoxicity.
Days 0-2 (0-48 hours after last methadone dose)	Withdrawal managed with short-acting hydromorphone.
~60 hours after last methadone dose	Moderate opioid withdrawal symptoms developed.
~60-76 hours after last methadone dose	Buprenorphine initiation intentionally delayed by the patient to minimize the risk of precipitated withdrawal.
Post-induction (day 3-4)	Transient worsening of withdrawal symptoms following buprenorphine initiation.
~48 hours after buprenorphine initiation	Withdrawal symptoms stabilized.
Discharge (approximately day 5)	Discharged on maintenance buprenorphine with outpatient follow-up arranged.

Therapeutic intervention

Based on shared decision-making, methadone was no longer considered an option and was not restarted during hospitalization. She was offered hydromorphone every two hours for short-term management of withdrawal symptoms. Hydromorphone was discontinued 48 hours after her last methadone dose. Understanding the risk of precipitated withdrawal, she voluntarily delayed initiation of buprenorphine for an additional 16 hours.

Outcomes

The patient's withdrawal symptoms improved substantially within 48 hours of buprenorphine initiation, and she was discharged 72 hours after the first buprenorphine dose with outpatient follow-up for ongoing buprenorphine management and treatment.

## Discussion

Currently, the best available evidence for transitioning patients from methadone to buprenorphine recommends gradual methadone tapering followed by buprenorphine initiation [8]. There is also emerging evidence supporting buprenorphine microdosing while patients continue methadone treatment [12]. However, to date, there is very little evidence supporting complete cessation of high-dose methadone without tapering before buprenorphine initiation.

To our knowledge, this is the first case in the literature to document successful complete cessation of high-dose methadone followed by transition to buprenorphine. This report is limited by its retrospective nature. Although key clinical events were documented, the authors no longer had access to the complete medical record during manuscript preparation and therefore could not report serial electrocardiograms (ECGs), detailed medication administration records, serial laboratory values, or standardized withdrawal assessments. These limitations reduce the level of clinical detail but do not alter the overall clinical course or rationale for management. In this case, the severity of the patient's methadone-associated adverse effects was life-threatening. Although hypokalemia may have contributed to the patient's QTc prolongation, the marked degree of QTc prolongation and the occurrence of ventricular arrhythmia in the setting of chronic high-dose methadone therapy are consistent with previously reported methadone-associated cardiotoxicity.

As a result of her cardiac event, the patient missed her morning dose of methadone. Following shared decision-making among the patient, the psychiatry team, and the primary team, methadone was discontinued, and she was started on short-acting hydromorphone as needed for withdrawal symptoms. After developing clinically significant opioid withdrawal, she was transitioned to buprenorphine and subsequently experienced stabilization of her withdrawal symptoms during the remainder of her hospitalization.

Although the conclusions that can be drawn from this case are limited, it suggests that future studies should investigate the safety and utility of complete methadone cessation without tapering before buprenorphine initiation. We highlight the importance of shared decision-making and establishing clear expectations regarding potential risks and adverse effects for both patients and treating teams. Although complete methadone cessation cannot be recommended routinely, this case suggests that it may be a feasible transition strategy in carefully selected clinical circumstances.

Current practice guidelines suggest tapering methadone to 40 mg daily before transitioning to buprenorphine. This case suggests that, in carefully selected patients for whom methadone becomes medically contraindicated, transition to buprenorphine without a preceding taper may be feasible under close inpatient monitoring. However, conclusions regarding safety, efficacy, or generalizability cannot be drawn from a single case, and additional studies are needed to better define appropriate patient selection and optimal transition strategies.

## Conclusions

In situations where methadone becomes medically contraindicated, abrupt methadone cessation followed by carefully timed buprenorphine initiation may represent a feasible management approach in select medically emergent situations. Long-term outcomes remain unknown, and additional prospective studies are needed before this approach can be recommended more broadly. This case highlights the importance of shared decision-making, close monitoring, and symptomatic support when managing urgent transitions from high-dose methadone to buprenorphine.

## References

[1] (2026). Methadose [package insert]. Food and Drug Administration. https://www.accessdata.fda.gov.

[2] (2026). Methadone hydrochloride [package insert]. Food and Drug Administration. https://www.accessdata.fda.gov.

[3] Enns B, Guerra-Alejos BC, Min JE, Carter A, Siebert U, Nosyk B (2025). Population-level health benefits and harms associated with buprenorphine/naloxone vs methadone. JAMA Netw Open.

[4] Nosyk B, Min JE, Homayra F (2024). Buprenorphine/naloxone vs methadone for the treatment of opioid use disorder. J Am Med Assoc.

[5] Chou R, Weimer MB, Dana T (2014). Methadone overdose and cardiac arrhythmia potential: findings from a review of the evidence for an American Pain Society and College on Problems of Drug Dependence clinical practice guideline. J Pain.

[6] Krantz MJ, Palmer RB, Haigney MC (2021). Cardiovascular complications of opioid use: JACC state-of-the-art review. J Am Coll Cardiol.

[7] Paknahad MH, Soleimani A, Baharlouei Yancheshmeh F, Adib-Hajbagheri P, Amerizadeh A, Karimi R (2025). QTc prolongation and torsades de pointes (TdP) in individuals undergoing methadone maintenance treatment (MMT): a systematic review and meta-analysis. Medicine (Baltimore).

[8] Funk MC, Nash S, Smith A (2022). Treatment of opioid use disorder in the general hospital. Am J Psychiatry.

[9] Chou R, Cruciani RA, Fiellin DA (2014). Methadone safety: a clinical practice guideline from the American Pain Society and College on Problems of Drug Dependence, in collaboration with the Heart Rhythm Society. J Pain.

[10] Becker WC, Seal KH, Nelson DB (2025). Buprenorphine, pain, and opioid use in patients taking high-dose long-term opioids: a randomized clinical trial. JAMA Intern Med.

[11] Dowell D, Ragan KR, Jones CM, Baldwin GT, Chou R (2022). CDC clinical practice Guideline for Prescribing Opioids for Pain - United States, 2022. MMWR Recomm Rep.

[12] Englander H, Thakrar AP, Bagley SM, Rolley T, Dong K, Hyshka E (2024). Caring for hospitalized adults with opioid use disorder in the era of fentanyl: a review. JAMA Intern Med.

[13] Casadonte PP (2024). Transfer from methadone to buprenorphine. Am J Addict.

